# Androgen Receptors Promote Oxidative Phosphorylation and Resistance to Palmitate Lipotoxicity in ER-Mutant Breast Cancer

**DOI:** 10.1210/endocr/bqaf168

**Published:** 2025-11-11

**Authors:** Dane T Sessions, Dillon P Boulton, Nicole S Spoelstra, M Cecilia Caino, Min Yu, Andrew Goodspeed, Jennifer K Richer

**Affiliations:** Department of Pathology, University of Colorado Anschutz Medical Campus, Aurora, CO 80045, USA; Department of Pathology, University of Colorado Anschutz Medical Campus, Aurora, CO 80045, USA; Department of Pathology, University of Colorado Anschutz Medical Campus, Aurora, CO 80045, USA; Department of Pharmacology, University of Colorado Anschutz Medical Campus, Aurora, CO 80045, USA; Department of Pharmacology and Physiology, University of Maryland School of Medicine, Baltimore, MD 21021, USA; Marlene and Stewart Greenebaum NCI Comprehensive Cancer Center, University of Maryland School of Medicine, Baltimore, MD 21201, USA; University of Colorado Cancer Center, University of Colorado Anschutz Medical Campus, Aurora, CO 80045, USA; Department of Biomedical Informatics, University of Colorado Anschutz Medical Campus, Aurora, CO 80045, USA; Department of Pathology, University of Colorado Anschutz Medical Campus, Aurora, CO 80045, USA

**Keywords:** breast cancer, androgen receptor, estrogen receptor, mitochondria, oxidative phosphorylation, fatty acid oxidation

## Abstract

Aromatase inhibitors (AI) are first-line therapy for postmenopausal women with estrogen receptor-expressing (ER+) breast cancer (BC). AI therapy effectively reduces recurrence and extends lifespan for patients with ER+ BC through long-term estrogen deprivation (LTED) resulting from inhibition of the enzyme aromatase that converts androgens to estrogens. However, up to 50% of ER+ BC recurs as AI-resistant metastatic disease within 10 years of diagnosis. AI-resistant BC upregulates androgen receptors (AR) and mitochondrial oxidative phosphorylation (OXPHOS) and requires OXPHOS and fatty acid oxidation (FAO). The liver and lung, common ER+ BC metastatic sites, have high abundance of the saturated fatty acid palmitate. We asked whether AR signaling regulates OXPHOS in the context of LTED. Using mutant ER-expressing MCF7 and T47D BC cell lines with AR antagonism via the anti-androgen enzalutamide and with shRNA knockdown, we demonstrate that AR supports cell growth, OXPHOS, FAO, and resistance to palmitate lipotoxicity. We identify AR as a positive regulator of the carnitine acyltransferase family enzyme CRAT that promotes OXPHOS capacity. These studies identify AR as pro-tumor in the LTED setting and as a therapeutic target for ER-mutant BC that develops under the selective pressure of AI therapy.

Breast cancer (BC) is the most common form of cancer in women, and two-thirds of primary breast tumors express estrogen receptor alpha (ER). First-line therapy for ER+ BC includes surgery and 5 to 10 years of endocrine therapy targeting ER directly or aromatase inhibitors (AI), which prevent estrogen biosynthesis. AI are more effective than tamoxifen at reducing BC recurrence-associated mortality in both postmenopausal women (1) and premenopausal women with ovarian suppression (2). AI block the conversion of androgens to estrogens via the enzyme aromatase but inadvertently increase circulating and intratumoral androgen levels (3-5). Although surgery and AI therapy are often effective in delaying the progression of ER+ BC, the 5- and 10-year recurrence rates are 9% and 19% (1), respectively, and the median survival with metastatic BC is less than 4 years (6). Approximately 40% of AI-resistant metastatic tumors demonstrate activating mutations in the ER ligand binding domain, the most common being Y537S and D538G (7-11). These mutations arise under the selective pressure of AI therapy, are detectable in circulating tumor cells and metastases, confer ligand-independent ER transcriptional activity, and permit cell survival and proliferation under long-term estrogen deprivation (LTED) engendered by AI therapy (12, 13). Androgen receptors (AR) are expressed in 80% to 90% of ER+ BC (14, 15). AR protein is often higher in metastatic compared to primary tumors (16), is increased with duration of AI therapy (17), and is elevated in ER-mutant cell lines and patient-derived xenografts in the LTED context under which these mutations arise. The role of AR in ER+ BC is controversial and highly contextual. Some reports indicate that AR is a tumor suppressor (18, 19), while others demonstrate a tumor supportive role for AR, particularly when the percent of cells positive for AR in a tumor is more than 2 times that of ER, and in the context of tamoxifen and AI therapy resistance (20-24). Our previous studies demonstrate that AR is strongly upregulated with LTED conditions (simulating AI therapy) and anchorage-independent survival, and that AR activity supports metastasis (17, 20). Furthermore, LTED induces a greater increase in AR expression in ER-mutant BC than ER-wildtype (20).

Metabolic alterations acquired by BC cells resistant to standard ER-targeting and AI therapies continue to be investigated. Recently, mitochondrial oxidative phosphorylation (OXPHOS) and fatty acid oxidation (FAO) were identified as critical for resistance to AI and traditional ER-targeting endocrine therapies (25-29). Specifically, anti-estrogen therapy induces expression of mitochondrial biogenesis and FAO genes (30, 31) and increases OXPHOS capacity and sensitivity to FAO inhibition (31). Interestingly, the common BC metastatic sites, liver and lung, harbor a high frequency of *ESR1* mutant metastases. These sites have high interstitial concentrations of the long-chain saturated fatty acid (FA) palmitate, which is also the most abundant circulating FA (32). Palmitate abundance at BC metastatic niches increases with high-fat diet, and palmitate oxidation promotes BC metastasis (32). Palmitate is an important substrate for FAO but can also cause tumor cell apoptosis (33, 34) if there is insufficient FAO to prevent toxic accumulation of this FA.

Since anti-estrogen therapy increases BC OXPHOS capacity and reliance on OXPHOS and FAO, we reasoned that OXPHOS inhibition may reduce therapy-resistant ER-mutant BC. Targeting mitochondrial OXPHOS directly with electron transport chain (ETC) inhibitors has yielded disappointing efficacy, with dosage limited by high systemic toxicities, as recently reviewed (35). Therefore, therapies that inhibit OXPHOS by interfering with tumor cell–specific mechanisms of OXPHOS regulation may offer viable therapeutic opportunity. Because both AR expression and OXPHOS capacity and reliance increase under anti-estrogen conditions, we sought to assess whether AR promotes OXPHOS and FAO in ER-mutant BC that persists under LTED conditions. We hypothesized that AR supports AI-resistant BC by promoting OXPHOS and FAO and that AR inhibition might be beneficial in combination with selective ER degraders (SERD) upon AI resistance.

We found that AR inhibition decreased ER-mutant BC adherent and anchorage-independent cell proliferation under LTED conditions, supporting a general pro-tumor role for AR in this therapeutic context. Further, AR antagonism strongly inhibited OXPHOS of fatty acids and other substrates, promoted palmitate-induced apoptotic lipotoxicity, and completely abrogated growth under conditions that require OXPHOS for ATP synthesis. While the anti-androgen enzalutamide did not elicit a broad anti-OXPHOS transcriptomic signature, it negatively regulated key metabolic enzymes, including the acetyl- and acyl-CoA abundance regulator carnitine O-acetyltransferase (*CRAT*). Metabolomic analysis of anti-androgen treated cells revealed a signature consistent with OXPHOS inhibition, including increased abundance of most even-chain acylcarnitine species and decreased abundance of L-aspartate, argininosuccinate, and UDP-N-acetyl-D-glucosamine. We provide the first functional evidence of AR positively regulating CRAT expression, suggesting that AR promotes mitochondrial function via this enzyme. Thus, AR inhibition may reduce tumor OXPHOS and activate lipotoxicity of ER+ BC metastases to liver and lung.

## Materials and Methods

### Cell Culture

Previously described MCF7 and T47D cell lines with heterozygous CRISPR knock-in ER Y537S and D538G mutations were obtained from the Gertz lab (13). MCF7 and T47D cells were authenticated by STR profiling and subject to recurrent Mycoplasma testing. MCF7 cells were cultured in phenol red-free MEM (Gibco 51200038) supplemented with 2mM glutamine, 5% charcoal-stripped fetal bovine serum (FBS), 1nM insulin, 1× nonessential amino acids (Gibco 11140050), 1× penicillin/streptomycin (Gibco 15140122), and 1nM dihydrotestosterone (DHT). T47D cells were cultured in phenol red-free RPMI1640 (Gibco 11835030) supplemented with 5% charcoal-stripped FBS, 1× penicillin/streptomycin, and 1nM DHT. All cells were cultured in charcoal-stripped FBS-containing media for 3 months or longer before experimental use. Reagent-specific information includes 0.8mM BSA (Cayman Chemical, Cat 29556), 5mM BSA-Palmitate (Cayman Chemical, Cat 29558), 5mM BSA-Oleate (Cayman Chemical, Cat 29557), and enzalutamide (MedChemExpress, Cat HY-70002).

### shRNA Knockdown

pLKO lentiviral vectors containing non-targeting control (shCTR, Millipore Sigma SHC002), human AR-targeting sequences (Broad shRNA sequences TRCN0000003715 [sh15] and TRCN0000003717 [sh17]), and human CRAT-targeting sequencings (Broad shRNA TRCN0000419400 [sh00] and TRCN0000035495 [sh95]) were purchased from the University of Colorado Anschutz Medical Campus Cancer Center Functional Genomics Core Facility (RRID:SCR_021987). Lentivirus was generated in HEK 293T cells in hormone-free media. Lipofectamine 3000 was used for transfection. Transduced cells were selected in 1.25 ug/mL puromycin.

### CRAT Overexpression

plx304 empty vector (EV, RRID: Addgene_25890) and human CRAT ORF-containing (CRAT) vectors (ccsbBroad304_10748) were purchased from the University of Colorado Anschutz Medical Campus Cancer Center Functional Genomics Core Facility (RRID: SCR_021987). Lentivirus generation and cell transduction were performed identically to shRNA virus above. Transduced cells were selected in 5 ug/mL blasticidin.

### Western Blot

Cell pellets were lysed in RIPA buffer (25mM Tris pH 7.6, 150mM NaCl, 1% NP40, 1% sodium deoxycholate, 0.1% SDS) supplemented with protease/phosphatase inhibitors on ice and were sheared with insulin needles. Protein concentration was measured using BCA assay and 20 to 30 ug protein was loaded per well onto 10% acrylamide gels. Proteins were transferred to PVDF membranes. Membranes were blocked in 3% BSA-Tris-buffered saline with Tween-20 (TBST) for one hour. Primary antibodies were diluted in 3% BSA TBST and incubated on membrane overnight on a rocker at 4 °C. Blots were rinsed 3 times in TBST for 10 minutes each on a shaker. Secondary antibody was diluted in 3% BSA TBST and applied to membranes for 1 hour at room temperature on a rocker. Membranes were rinsed again 3 times before visualization using Licor Odyssey. Antibody specific information includes alpha tubulin (Sigma-Aldrich Cat# T5168, RRID: AB_477579), PARP (Cell Signaling Technology Cat# 9542, RRID: AB_2160739), AR (Cell Signaling Technology Cat# 5153, RRID: AB_10691711), FKBP5 (Cell Signaling Technology Cat# 8245, RRID: AB_10831198), CPT1A (Cell Signaling Technology Cat# 12252, RRID: AB_2797857), CD36 (Sigma-Aldrich Cat# HPA002018, RRID:AB_1078464), CRAT (Proteintech Cat# 15170-1, RRID: AB_2229978), and anti-Mitochondria (Invitrogen MA5-12017, RRID: AB_10983622).

### RNA/cDNA Preparation

RNA was isolated using Qiagen RNeasy Mini kits and quantitated on a NanoDrop 2000. Using Quantabio qScript cDNA synthesis kit, 1 μg cDNA was prepared per manufacturer protocol. After synthesis, cDNA was diluted to 10 ng/μL in water and frozen.

### Quantitative Polymerase Chain Reaction

Quantitative polymerase chain reaction (qPCR) was performed using PowerUp SYBR Green Master Mix (Thermofisher Cat# 4309155). Reactions were run with 10 ng cDNA and 0.5μM primer per reaction in 20 μL per well in 96-well plates and run in technical and biological triplicate. Relative mRNA levels were calculated using the 2^−ΔΔCt^ method with 18S as a control target. Primer sequences: CRAT-F:AAGGCTCTAGCAAGGACCCA, CRAT-R: CAGAGGCTTCACCACGGTC, 18S-F: GTAACCCGTTGAACCCCAATT, 18S-R: CCATCCAATCGGTAGTAGCG.

### RNA Sequencing

RNA purity, quantity, and integrity was determined with NanoDrop (ThermoFisher Scientific) and TapeStation 4200 (Agilent) analysis prior to RNA-seq library preparation. The Universal Plus mRNA-Seq library preparation kit with NuQuant was used (Tecan) with an input of 200 ng of total RNA to generate RNA-seq libraries. Paired-end sequencing reads of 150 bp were generated on NovaSeq X (Illumina) sequencer at a target depth of 80 million paired-end reads per sample. Raw sequencing reads were de-multiplexed using bcl2fastq.

### RNA-seq Processing

RNA-seq data were processed using the nf-core RNA-seq pipeline (version 3.12.0). Briefly, Illumina adapters were removed using Cutadapt (version 3.4) as part of the trimgalore (0.6.7) package. Reads were aligned using STAR (2.7.9a) to the Ensembl human transcriptome (GRCh38 release 104) and quantified using Salmon (1.10.1). Raw data with counts by gene was generated using tximport on Salmon quantified data. Normalized data was generated to counts per million (36). Differential expression was calculated using the limma R package (37). Gene set enrichment analysis (GSEA) was performed using fold-change and the fgsea R package with Gene Ontology biological processes gene sets from the Molecular Signatures Database (38) which were downloaded using the msigdbr R package. Plotting utilized the ggplot2 R package. Raw and processed RNA-seq data was deposited in the Gene Expression Omnibus (GSE296966).

### Metabolomics

MCF7 Y537S cells were seeded at 500 000 cells per well in 6-well dishes and incubated with dimethyl sulfoxide (DMSO) or 30μM enzalutamide for 72 hours. Media was changed to fresh media, including 10μM BSA-palmitate to provide fatty acid for FAO. Cells were harvested and counted after 24 hours in palmitate-containing media. Cell samples were diluted with a chilled extraction solution of methanol, acetonitrile, and water (50:30:20, v/v/v) at a ratio of 1 mL per 2 million cells. After vigorously vortexing at 4 °C for 30 minutes, samples were centrifuged at 12 000*g* for 10 minutes at 4 °C. The resulting supernatants were collected for metabolomics analysis by ultra-high-pressure liquid chromatography–mass spectrometry (UHPLC-MS). Metabolomics analysis was performed using a Vanquish UHPLC system (Thermo Fisher Scientific) coupled to an Orbitrap Exploris 120 mass spectrometer (Thermo Fisher Scientific). Samples (10 µL) were injected onto a 2.1 × 100 mm Waters Acquity BEH C18 column with 1.7-µm particle size and separated using a 5-minute reversed-phase gradient, as previously described (39).The mass spectrometer was run independently in both negative and positive ion modes, acquiring full MS scans over an m/z range of 65 to 975 at a resolution of 120 000. Source conditions included a sheath gas flow of 50 arbitrary units (Arb), auxiliary gas flow of 10 Arb, and spray voltages of 3 kV (negative mode) and 3.4 kV (positive mode). The instrument was calibrated prior to analysis using the Easy-IC internal standard (Thermo Fisher Scientific). Sample run order was randomized. Technical replicates were injected at the beginning, middle, and end of each sequence to assess analytical reproducibility and quality control. Raw files were converted to .mzXML format using RawConverter and analyzed with El-Maven (Elucidata) using the KEGG database for metabolite identification and peak integration, as previously described (40). Metabolomics data for this study is available at the NIH Common Fund's National Metabolomics Data Repository (NMDR) website, the Metabolomics Workbench, https://www.metabolomicsworkbench.org, where it has been assigned Study ID ST003967.

### CellTiter-Glo Assay

CellTiter-Glo (CTG) reagent (Promega, Cat G7571) was prepared according to manufacturer recommendations. Cells were pretreated with DMSO or 30μM enzalutamide for 72 hours before seeding at 20 000 cells in 100 μL per well in 96-well plates. Assay-specific compounds were added when seeded. Then 100 μL CTG reagent was added per well 24 hours after cell seeding. Cells were incubated with CTG reagent for 12 minutes at room temperature on a shaker before luminescence measurement by plate reader.

### Incucyte Cell Proliferation Assay

Cells were seeded at 5000 cells in 200 μL media per well in 96-well plates. For acetoacetate media assays, cells were seeded at 10 000 cells in 200 μL media per well in 96-well plates. Acetoacetate media was prepared using no phenol red, no glutamine, no glucose DMEM (Thermofisher Cat#A1443001) supplemented with 2mM glutamine, 5% charcoal-stripped FBS, 1× nonessential amino acids, 1× penicillin/streptomycin, 1nM insulin, 1nM DHT, and 5.5mM lithium acetoacetate (Fisher Cat# A14781G). Wells were imaged once per day by Incucyte whole-well imaging and confluence compared to Day 0 was calculated using Incucyte software. Image analysis parameters: Cell Segmentation: 0.5, Whole well keep-out: 100.

### CellTOX Assay

Palmitate lipotoxicity and acetoacetate toxicity assays were seeded at 20 000 cells per black-walled 96-well plates in 200 μL media containing indicated compounds and 1× CellTOX Green reagent (Promega Cat# G8741). Acetoacetate media formulation can be found above under Incucyte Cell Proliferation Assay methods. Palmitate lipotoxicity assays were incubated for 24 hours before fluorescence reading. Acetoacetate toxicity assays were incubated 48 hours before reading. Fluorescence was measured on a Synergy Neo 2 plate reader with excitation/emission spectra (bandwidth filter) of excitation 485(9), emission 530 (20). After reading, cells were permeabilized with 0.5% Triton X-100 for 30 minutes at room temperature then measured again for normalization to total possible cell fluorescence per well.

### Soft Agar Colony Formation Assay

Noble agar (BD Biosciences Cat# 214230) was prepared in phosphate-buffered saline (PBS) to 1.5% and autoclaved. The bottom layer was prepared at 0.5% agar by mixing with cell media and 1 mL was dispersed per 12-well well and allowed to solidify. Then 5000 cells per well were seeded in 0.3% agar diluted in media in 1 mL per well. Wells were provided with 1 mL media shortly after upper agar layer solidified. Wells were then provided 0.25 mL fresh media twice weekly for a total assay duration of 3 weeks. After 3 weeks, cells were stained with 0.25 mL of 1 mg/mL Nitroblue tetrazolium in PBS per well, incubated overnight, and imaged the next day on a Biorad Chemidoc (RRID:SCR_021693). Colonies were quantified using FIJI (RRID:SCR_002285).

### Seahorse FAO Assay

Cells were pretreated with DMSO or indicated compounds for 72 hours before seeding in 80 μL nutrient-limited media with DMSO or enzalutamide (Gibco A1443001 supplemented with 0.5mM glucose, 1mM glutamine, 0.5mM carnitine, 1% charcoal-stripped FBS, 1nM DHT) per well in Seahorse 96-well microplate wells. MCF7 cells were seeded at 25 000 cells per well, and T47D cells were seeded at 30 000 cells per well. One day after seeding, nutrient-limited media was manually aspirated and replaced with manufacturer-recommended FAO buffer supplemented with 2.5mM glucose, 0.5mM carnitine, 5mM HEPES pH 7.4, and 200μM BSA-palmitate. Indicated wells also contained either DMSO or 40μM etomoxir. Cells were placed in a non-CO_2_ 37 °C incubator for 1 hour before assay start. Final concentrations of inhibitors were oligomycin (1μM), FCCP (2μM), rotenone (1μM), and antimycin A (1μM). Reagent-specific information includes Seahorse XFe96/XF Pro FluxPak (Agilent, Cat 103793-100), 5 mM BSA-Palmitate (Cayman Chemical, Cat 29558), FCCP (Cayman Chemical, Cat 15218), Oligomycin (Cayman Chemical, Cat 11341), L-Carnitine (Sigma, Cat C0283), and Etomoxir Sodium (Sigma, Cat E1905). Average values for each phase (resting, post-oligomycin, post-FCCP, and post-Antimycin A/Rotenone) were calculated. Maximal mitochondrial oxygen consumption capacity (OCR) was calculated as (Post-FCCP)_Avg_ − Post(Antimycin A/Rotenone)_Avg_. Substrate-specific maximal OCR values were calculated for DMSO and enzalutamide groups as: “FAO” = OCR_DMSO_ − OCR_Etomoxir_, and “Other” = OCR_Etomoxir_.

### Seahorse Acetoacetate Mitochondrial Stress Test

MCF7 cells were pretreated with DMSO or 30μM enzalutamide for 72 hours before seeding 25k cells in 80μL media with DMSO or enzalutamide per well in Seahorse 96-well microplate wells. One day after seeding, media was manually aspirated and replaced with 180μL Seahorse XF Base Medium (Agilent Cat 103335-100) supplemented with 5mM HEPES, 2mM glutamine, 1mM sodium pyruvate, and 10mM lithium acetoacetate. Cells were placed in a non-CO_2_ 37 °C incubator for 1 hour before assay start. Final concentrations of inhibitors were oligomycin (1μM), FCCP (2μM), rotenone (1μM), and antimycin A (1μM). Maximum OCR was calculated as in FAO assays.

### Seahorse Glycolysis Stress Test

MCF7 cells were pretreated with DMSO or 30μM enzalutamide for 72 hours before seeding 25k cells in 80 μL media with DMSO or enzalutamide per well in Seahorse 96-well microplate wells. One day after seeding, media was manually aspirated and replaced with 180 μL Seahorse XF Base Medium supplemented with 5mM HEPES and 2mM glutamine. Cells were placed in a non-CO_2_ 37 °C incubator for 1 hour before assay start. Final concentrations of injected compounds were glucose (10mM), oligomycin (1μM), and 2-deoxyglucose (50mM, Cayman Chem Cat 14325). Maximum ECAR was calculated as (Post-Oligo)_Avg − _(Post-2-deoxyglucose)_Reading 3_ for each well. Reading 3 was selected for the Post-2-deoxyglucose values instead of the average as the effect of 2-deoxyglucose was incomplete during the first and second readings, as visible in the ECAR curves.

### Immunofluorescence

250 000 cells were seeded onto cover slips in 6-well plates in media containing DMSO or 30μM enzalutamide and grown for 4 days. Media was aspirated, and cells were rinsed in PBS and fixed in 10% formalin for 15 minutes at room temperature. Cells were rinsed 3 times in PBS and permeabilized in 0.1% Triton X-100 PBS for 5 minutes at room temperature. Cells were rinsed in PBS 3 times and blocked in 5% normal goat serum 0.3M glycine PBS for 30 minutes at room temperature. Slips were incubated in blocking buffer containing anti-MTC02 antibody (Invitrogen Cat# MA5-12017, RRID:AB_10983622) diluted at 1:500 overnight at 4 °C. The next day, primary antibody solution was removed, and cells were rinsed 3 times in PBS. Secondary antibody was diluted 1:500 in blocking buffer, added to cells, and incubated at room temperature for 1 hour. Cells were rinsed 3 times in PBS and counterstained with DAPI for 10 minutes. Cells were mounted onto slides with Prolong Glass. Slides were imaged on a Zeiss LSM780, and mitochondrial abundance was quantified using MitoGenie (mitogenie.com) (41).

### Immunohistochemistry

Cells were fixed in 10% neutral buffered formalin for 30 minutes., washed in PBS before and after fixation, and embedded in Histogel (Epredia Cat HG-4000-012) prior to tissue processing and paraffin embedding by the CU Anschutz Histology Shared Resource. Paraffin sections (5 μm) were placed on charged slides, baked at 60 °C for 1 hour, and deparaffinized in a series of xylenes and graded ethanols. Heat-induced epitope retrieval was performed using a Biocare Medical Decloaker with 10mM Tris/1mM EDTA pH9.0 buffer at 110 °C for 10 minutes. Sections were blocked using 3% hydrogen peroxide and TBST was used for both permeabilizing and washing slides. Primary antibody CRAT (Proteintech Cat 15170-1-AP) was applied for 1 hour and detected using the ImmPRESS® HRP Goat Anti-Rabbit IgG Polymer Detection Kit, (Vector Laboratories cat MP-7451) followed by Vina Green chromagen detection (Biocare Medical, cat# BRR807A) according to the manufacturer's instructions. Pre- and post-treatment matched tissue sections from 5 patients per treatment group at baseline (core needle biopsies) and 4 months post-treatment (surgical excision biopsies at time of surgery) from a randomized phase 2 trial (NCT02955394; COMIRB 16-1042) of the ER degrader fulvestrant with or without enzalutamide that were previously stained for AR were additionally stained for CRAT. Slides were soaked in xylene to remove coverslips and stained for CRAT using the procedure described above. IM3 images (n = 3 20× fields per tumor of field size 669 μm × 500 μm) were taken on the Vectra 3 Pathology Imaging System (Akoya Biosciences) for n = 5 patients per treatment arm. Images were segmented for tissue (tumor/stroma) and cells (nucleus/cytoplasm) before phenotyping for AR and CRAT expression using inForm 2.6 and Phenoptr software (Akoya Biosciences). Violin plots stratified by treatment arm (Combo vs Fulvestrant) and time (baseline, BL vs surgery) were generated. Group averages (horizontal lines) and Z-test *P* values were estimated using a linear mixed-effects model.

### NAD/NADH-Glo Assay

NAD/NADH-Glo reagent (Promega, Cat G9017) was prepared according to manufacturer recommendations. Cells were pretreated with DMSO or 30μM enzalutamide for 72 hours before seeding at 100 000 cells per well in 2 mL media with DMSO or enzalutamide in 12-well plates. After 24 hours, cells were harvested, resuspended in PBS, and counted. For each well, 5000 cells were used for NAD+ and NADH measurement. Cells were lysed in 0.2N NaOH + 0.05% Triton X-100 for 3 minutes before loading 50 μL cell lysate per well in white-walled 96-well plates. Then 25 μL 0.4N HCl was added to all NAD+ wells before incubating all wells at 60 °C for 15 minutes. Plate was equilibrated to room temperature for 10 minutes before adding 25 μL 0.5M Tris base to NAD+ wells and 50 μL HCl/Tris solution to NADH wells. Then 100 μL NAD/NADH-Glo reagent was added to all wells and the plate was incubated at room temperature in the dark for 1 hour before luminescence measurement. NAD+/NADH ratio was calculated by NAD+ luminescence/NADH luminescence.

### CBioPortal Correlations

AR and CRAT expression were queried in TCGA Firehose Legacy Invasive Breast Carcinoma datasets, limiting samples to ER+ by IHC (https://bit.ly/4jb84tE). AR and CRAT mRNA and protein expression were exported and plotted in Graphpad Prism.

### Statistical Analysis

Statistics were performed, and charts were generated, using Graphpad Prism version 10.4.1 (RRID: SCR_002798). Specific statistical tests and measures of variation are indicated in figure legends. False discovery rate (FDR) < 0.05 was defined as statistically significant for RNA-seq and metabolomics data.

## Results

### AR Promotes ER-Mutant BC Growth Under LTED Conditions

**Figure 1.**
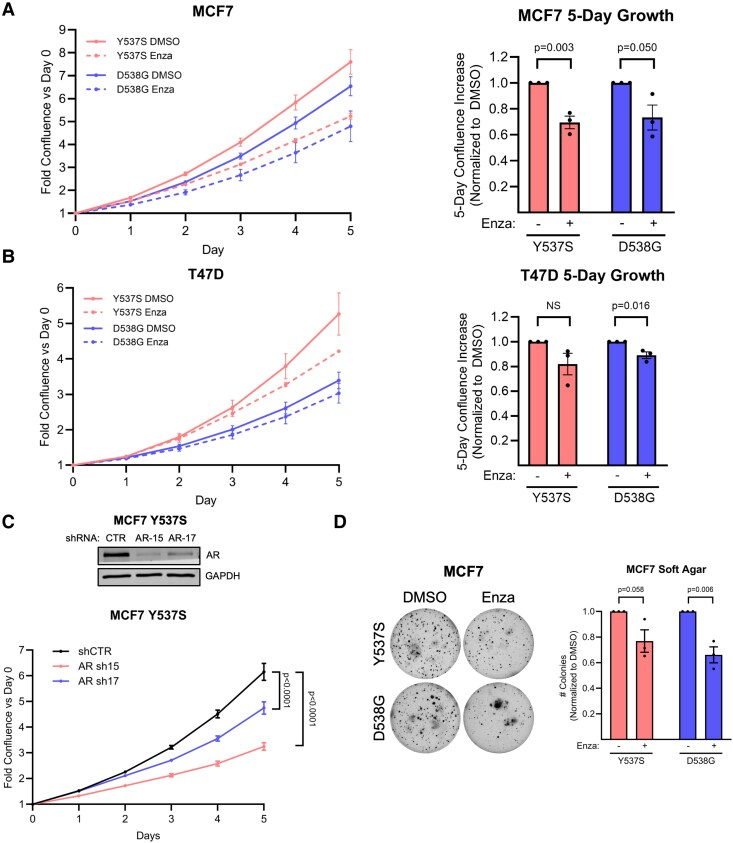
AR promotes ER-mutant BC growth under LTED. A-B, Adherent cell growth of DMSO- or enzalutamide-treated (30μM) ER-mutant MCF7 (A) and T47D (B) cells measured by fold confluence vs day 0 by Incucyte. Mean ± SEM of 3 independent experiments with each experiment contributing one data point. Quantitation of Day 5 confluence vs Day 0 normalized to DMSO with 2-tailed unpaired Student's *t* test. C, Western blot confirmation of AR knockdown by shRNA in MCF7 ER Y537S cells and adherent cell growth measured by fold confluence vs Day 0 by Incucyte. N = 6 technical replicates per experiment, representative of 2 independent experiments. One-way ANOVA + Dunnett's multiple comparisons test. D, Representative soft agar colony formation of MCF7 ER-mutant cells with colony number quantitation normalized to DMSO. Mean ± SEM of 3 independent experiments. 2-tailed unpaired Student's *t* test.

To model the hormonal milieu of ER-mutant BC in women on AI therapy, we performed prolonged (>3 months) culture of CRISPR heterozygous knock-in ER Y537S or D538G MCF7 and T47D cells (13) in phenol red-free media with charcoal-stripped FBS and 1nM dihydrotestosterone (DHT). Since the role of AR in ER-positive BC is controversial and context-dependent, we first sought to identify whether AR inhibition affects adherent ER-mutant BC growth under AI-mimicking conditions. We found that enzalutamide significantly inhibited, but did not completely suppress, proliferation of ER-mutant MCF7 Y537S and D538G cells and T47D D538G cells in media with standard (5.5mM) glucose availability, whereas T47D Y537S cells demonstrated a similar trend but did not reach statistical significance (Fig. 1A and 1B). As enzalutamide was recently reported to also inhibit ER (42), we confirmed that growth inhibition from enzalutamide was consistent with inhibition of AR using AR knockdown as an alternative approach (Fig. 1C). We also assessed whether AR inhibition affected anchorage-independent growth and found that enzalutamide significantly inhibited soft agar colony formation capacity of MCF7 D538G cells and demonstrated a similar effect trend in MCF7 Y537S cells that did not reach statistical significance (Fig. 1D). These studies broadly identified AR as a tumor-promoting receptor in ER-mutant BC under conditions simulating AI therapy.

### AR Promotes ER-Mutant BC OXPHOS and FAO Capacity Under LTED Conditions

**Figure 2.**
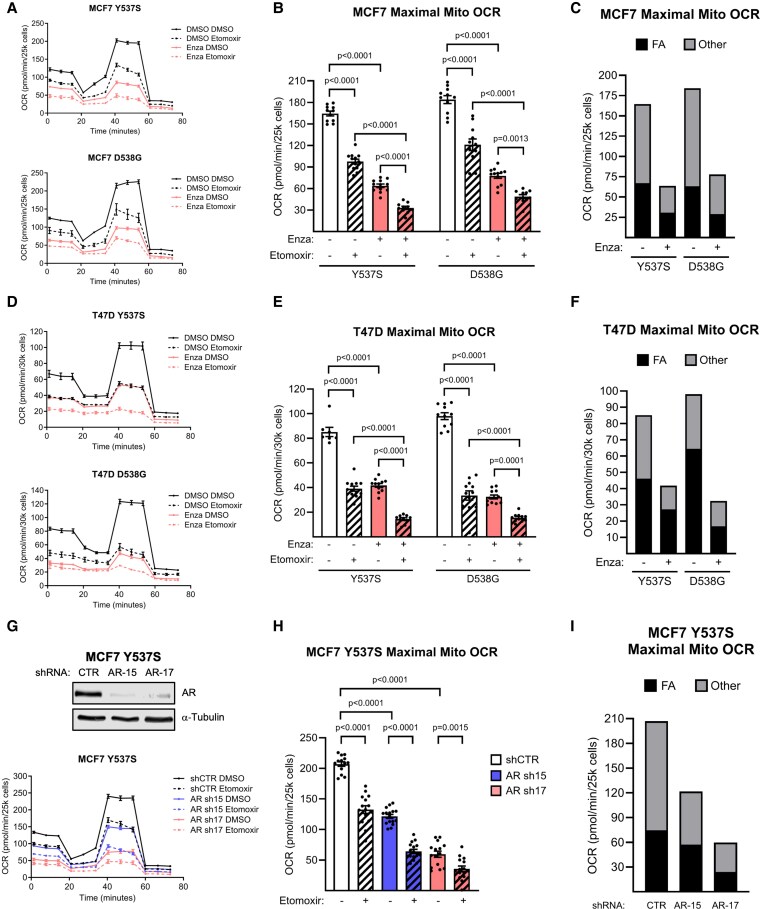
AR promotes ER-mutant BC OXPHOS and FAO under LTED. A-C, Seahorse FAO assay curves (A), total maximal mitochondrial OCR (B), and substrate-dependent OCR (C) in DMSO- and enzalutamide-treated (30μM, 4 days) MCF7 ER-mutant cells. D-F, Seahorse FAO assay curves (D), total maximal mitochondrial OCR (E), and substrate-dependent OCR (F) in DMSO- and enzalutamide-treated (30μM, 4 days) T47D ER-mutant cells. G-I, Western blot confirmation of AR knockdown and Seahorse FAO assay curves (G), total maximal mitochondrial OCR (H), and substrate-dependent OCR (I) in AR knockdown MCF7 ER Y537S cells. Mean ± SEM of 8-16 replicates. One-way ANOVA + Sidak's multiple comparisons test. FA = fatty acid oxidation. Other = oxidation due to non-FA substrates.

Since OXPHOS and FAO have been recently reported as upregulated under prolonged anti-estrogen therapy and critical to anti-estrogen-resistant BC persistence, we performed Seahorse FAO assays to assess whether AR promotes ER-mutant BC OXPHOS and FAO under AI therapy-mimicking conditions. We found that enzalutamide inhibited total oxygen consumption capacity (OCR) of MCF7 (Fig. 2A-2C) and T47D (Fig. 2D-2F) cells. Enzalutamide inhibited oxidation of both fatty acids and non-fatty acid substrates. We confirmed this phenotype using AR knockdown and observed similar results to the effect of enzalutamide (Fig. 2G-2I).

Potential mechanisms by which AR antagonism could inhibit OXPHOS include reduction of cellular mitochondrial content or induction of ETC dysfunction. We therefore assessed mitochondrial abundance by Western blot and immunofluorescence microscopy and found that 4 days of enzalutamide treatment did not affect mitochondrial content in ER-mutant BC cells (Supplementary Fig. S1A and S1B (43 )). Severe ETC dysfunction causes general OXPHOS inhibition and inability to efficiently regenerate NAD+ from NADH. We found that enzalutamide did not affect the NAD+/NADH ratio (Supplementary Fig. S1C (43)), suggesting that the ETC is not severely inhibited under anti-androgen therapy. Since others have reported that anti-estrogen therapy increases expression of the long-chain fatty acid carnitine acyltransferase CPT1A required for FA transport inside mitochondria (31), as well as the cell surface fatty acid transporter CD36 (44) in BC, we assessed whether enzalutamide affects either CPT1A or CD36 expression. We found that suspension culture, which strongly upregulates AR and its positive target gene FKBP5, also increased CD36 and CPT1A, but that these 2 proteins were unaffected by enzalutamide treatment at least at this dose and timepoint (Supplementary Fig. S1D and S1E (43)). These data demonstrate that enzalutamide causes OXPHOS inhibition independent of substrate, mitochondrial content, severe ETC dysfunction, or effect on the key FAO proteins CPT1A and CD36.

### AR Antagonism Promotes ER-Mutant BC Palmitate Lipotoxicity

**Figure 3.**
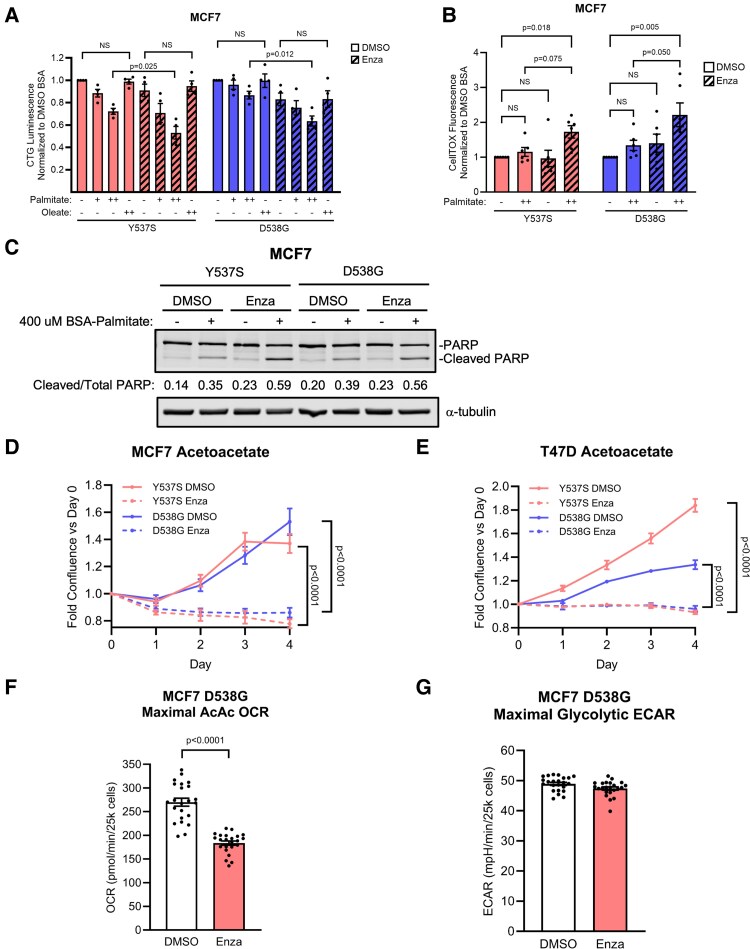
AR inhibition promotes palmitate lipotoxicity and inhibits OXPHOS-dependent growth in ER-mutant BC under LTED. A-B, CellTiter-Glo (CTG) analysis of energetic viability (A) and CellTOX analysis of cell death (B) in DMSO- or enzalutamide-treated (30μM) MCF7 ER-mutant cells treated with BSA, BSA-palmitate or BSA-oleate and normalized to DMSO + BSA treatment group. (−) = BSA only, (+) = 200μM, (++) = 400μM. Mean ± SEM of 4 to 6 independent experiments. One-way ANOVA + Sidak's multiple comparisons test. C, Western blot of PARP cleavage as a readout of apoptosis in DMSO- or enzalutamide-treated (30μM) MCF7 ER-mutant cells treated with BSA only or 400μM BSA-palmitate. Representative of 2 independent experiments. D-E, Adherent cell growth of DMSO- or enzalutamide-treated ER-mutant MCF7 (D) or T47D (E) cells in glucose-free acetoacetate-containing media measured by fold confluence vs Day 0 by Incucyte. N = 6 technical replicates per experiment, representative of 2 independent experiments. 2-tailed unpaired Student's *t* test. F, Maximal mitochondrial OCR of DMSO- or enzalutamide-treated (30μM, 4 days) MCF7 D538G cells in glucose-free acetoacetate (AcAc)-containing media measured by Seahorse mitochondrial stress test. Mean ± SEM of 23 replicates. 2-tailed unpaired Student's *t* test. G, Maximal glycolytic ECAR of DMSO- or enzalutamide-treated (30μM, 4 days) MCF7 D538G cells by Seahorse glycolysis stress test. Mean ± SEM of 23 replicates.

Recent work demonstrated that palmitate is highly abundant in the liver and lung, which are common ER+ BC metastatic sites (32). Palmitate is the most abundant circulating saturated FA and can undergo FAO inside of mitochondria to form acetyl-CoA; however, palmitate accumulation can also be cytotoxic and cause cell death by inducing apoptosis (33, 34). Conversely, the unsaturated fatty acid oleic acid is known to be generally non-lipotoxic (45). We reasoned that metastatic tumor cells in the lung and liver will encounter palmitate and may use palmitate as an energy source but must also be able to metabolize it to avoid excessive accumulation and lipotoxicity. Since we found AR inhibition decreased OXPHOS and FAO in ER+ BC, we hypothesized that AR inhibition may also sensitize these cells to palmitate-mediated lipotoxicity. We found that enzalutamide impaired palmitate-treated, but not oleic acid-treated, ER+ BC energetic viability (Fig. 3A) and increased palmitate-mediated cell death (Fig. 3B). We further found that enzalutamide promoted palmitate-mediated apoptosis (Fig. 3C). This indicates that although AR inhibition sensitizes ER-mutant BC to palmitate-induced apoptosis, it does not universally sensitize to lipid exposure.

Recently, identifying potential effects of dietary intervention, specifically the ketogenic diet, on ER-positive BC treatment efficacy has garnered interest (30). The ketogenic diet restricts carbohydrates and promotes ketone body circulation. Ketone bodies such as acetoacetate undergo ketolysis to form acetyl-CoA inside mitochondria, and therefore ATP from acetoacetate must be derived by mitochondrial OXPHOS. Conversely, carbohydrate-containing diets and standard culture media provide glucose that allows cytoplasmic ATP synthesis via glycolysis even if OXPHOS is dysfunctional. Therefore, we asked whether the effects of enzalutamide on cell growth may be magnified by culturing cells in glucose-free acetoacetate-containing media such that only cells with intact OXPHOS survive and grow. We found that enzalutamide completely abrogated ER-mutant BC growth (Fig. 3D and 3E) and promoted cell death in glucose-free acetoacetate media (Supplementary Fig. S2A and S2B, (43)). We then determined whether enzalutamide affects acetoacetate oxidation or glycolytic capacity and found that enzalutamide inhibited acetoacetate oxidation (Fig. 3F and Supplementary Figure S2C (43)), but not glycolysis (Fig. 3G and Supplementary Fig. S2D (43)). These findings may explain why the effect of AR inhibition of cell proliferation was more profound in the presence of acetoacetate compared to glucose-containing culture and highlights how some aspects of AR function (and effects of inhibiting this receptor) are more evident under conditions that require OXPHOS.

### Enzalutamide Inhibits Key Metabolic Enzyme Expression and Induces an OXPHOS Inhibition Metabolic Signature

**Figure 4.**
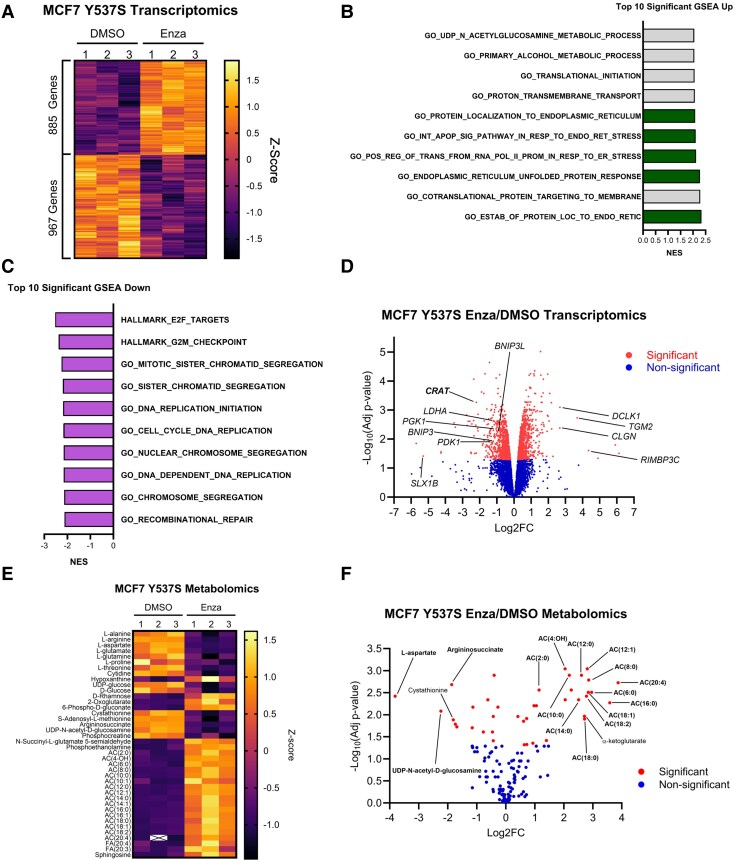
AR inhibition results in downregulation of key metabolic enzymes and promotes a metabolic signature of OXPHOS inhibition in ER-mutant BC under LTED. A, Heatmap displaying CPM Z-scores of differentially expressed genes in DMSO- vs enzalutamide-treated (30μM, 4 days) MCF7 Y537S cells. B-C, GSEA demonstrating top 10 statistically significantly activated (B) and inactivated (C) pathways by NES under enzalutamide treatment. D, Volcano plot of gene expression from (A) with select genes annotated. E, High Throughput Global Metabolomics-derived Z-score heatmap of differentially abundant metabolites in DMSO- vs enzalutamide-treated (30μM, 4 days) MCF7 Y537S cells. F, Volcano plot of gene expression from (E) with select metabolites annotated. Significance defined by FDR <0.05.

To assess whether AR inhibition affects the transcriptomic landscape of ER-mutant BC in a manner that could explain the role of this steroid hormone receptor on OXPHOS, we performed RNA sequencing on MCF7 Y537S cells treated with DMSO or enzalutamide. We found that enzalutamide treatment significantly affected the expression of 1853 genes and caused both increases and decreases in gene expression consistent with the ability of AR to act as a gene specific transcriptional activator or repressor (Fig. 4A). Pathway analysis using GSEA revealed that enzalutamide significantly increased activation of endoplasmic reticulum pathways (Fig. 4B), and downregulated cell cycle pathways (Fig. 4C). Although OXPHOS or FAO were not among the top significantly downregulated pathways, numerous genes involved in carbon metabolism, mitochondrial metabolism, or mitochondrial dynamics, including *CRAT*, *LDHA*, *PGK1*, *PDK1*, *BNIP3*, and *BNIP3L,* were significantly decreased by 2-fold or greater (Fig. 4D). Interestingly, all mitochondrial DNA (mtDNA)-encoded transcripts that contributed to the “proton transmembrane transport” Gene Ontology (GO) term demonstrated a statistically significant but small-magnitude increase with enzalutamide treatment (Supplementary Fig. S3A, (43)), indicating that enzalutamide does not suppress OXPHOS by inhibiting mtDNA transcription and instead may suggest a mitochondrial gene transcription feedback loop when OXPHOS is inhibited by other means. To examine how enzalutamide affects metabolism of ER-mutant BC, we performed high throughput metabolomics on MCF7 ER Y537S cells treated with DMSO or enzalutamide. We found that enzalutamide-treated cells demonstrated both decreased abundance of many OXPHOS-dependent metabolites including L-aspartate, argininosuccinate, and UDP-N-acetyl-D-glucosamine and increased abundance of almost all even-chain acylcarnitine species (Fig. 4E and 4F), consistent with FAO dysfunction (46).

### AR Promotes CRAT Expression in Breast Cancer

**Figure 5.**
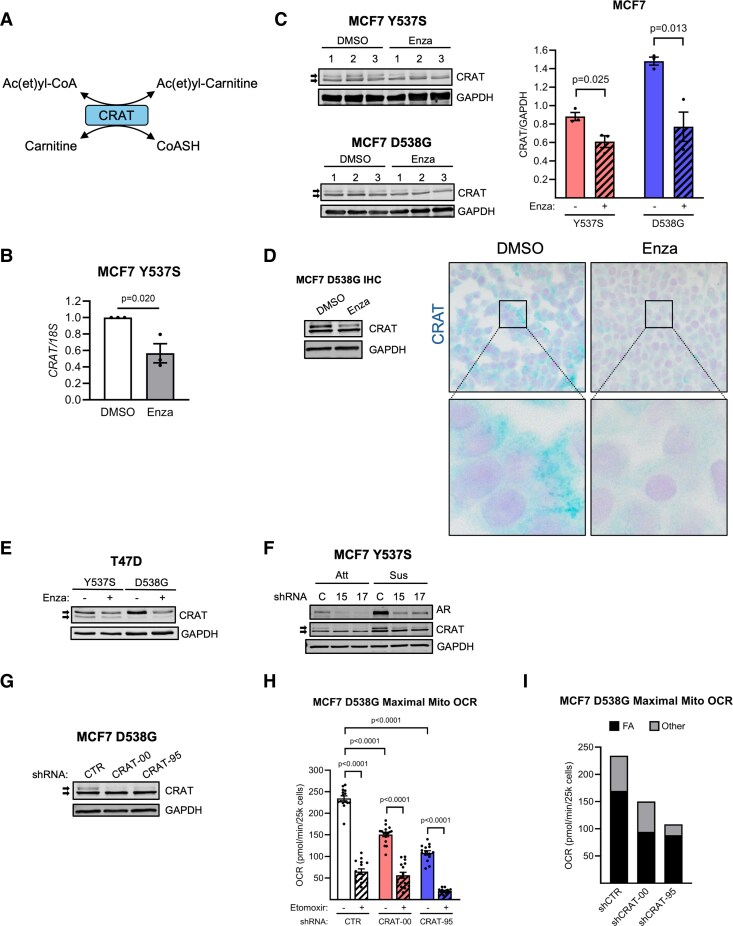
AR promotes CRAT expression and CRAT promotes OXPHOS in ER-mutant BC under LTED. A, Schematic of CRAT enzymatic activity demonstrating bidirectional synthesis of ac(et)yl-CoA and ac(et)yl-carnitine with carnitine and CoASH. B, qPCR measurement of *CRAT* mRNA abundance in DMSO- or enzalutamide-treated (30μM, 4 days) MCF7 ER Y537S cells used for RNA sequencing. Mean ± SEM of 3 independent replicates normalized to DMSO control. 2-tailed unpaired Student's *t* test. C, Western blot of CRAT in DMSO- or enzalutamide-treated (30μM, 3 days) ER-mutant MCF7 cells with densitometry quantitation. Mean ± SEM of 3 independent experiments. 2-tailed unpaired Student's *t* test. D, CRAT immunohistochemistry in MCF7 D538G cells treated with DMSO or 30μM enzalutamide for 4 days; 400× magnification. E, Western blot of CRAT in DMSO- or enzalutamide-treated (30μM, 7 days) ER-mutant T47D cells. F, Western blot of AR and CRAT in MCF7 ER Y537S AR knockdown cells after 5 days of attached (Att) or suspension (Sus) culture. G, Western blot of CRAT in MCF7 D538G CRAT knockdown cells. H-I, Seahorse FAO assay maximal mitochondrial (H) and substrate-dependent (I) OCR in MCF7 D538G CRAT knockdown cells. Mean ± SEM of 15 to 16 replicates. One-way ANOVA + Sidak's multiple comparisons test. Larger and smaller CRAT protein isoforms are labeled with arrows.

We decided to assess the potential role of carnitine O-acetyltransferase (*CRAT*) in enzalutamide-mediated OCR inhibition given that it had one of the greatest decreases in expression from enzalutamide treatment and is known to regulate OXPHOS and FAO. CRAT is a mitochondrial- and peroxisomal-targeted enzyme that catalyzes the reversible transfer of short-chain acyl or acetyl groups from Coenzyme A to carnitine to form acyl- or acetyl-carnitine species and liberating CoASH in the process (Fig. 5A). CRAT transfers acyl chains 2 (C2) to 10 (C10) carbons in length onto carnitine and forms carnitine conjugates with metabolites from branched chain amino acid and ketone metabolism (47). CRAT is critical to regulation of mitochondrial carbon metabolism and CoA stores, but CRAT depletion can inhibit OXPHOS in some cells (48) and promote OXPHOS in others (49). We found that enzalutamide inhibited ER-mutant BC *CRAT* mRNA by qPCR (Fig. 5B) and that CRAT protein demonstrated a doublet banding pattern, as others have reported (50, 51), that was decreased in MCF7 cells by 3 days of enzalutamide treatment (Fig. 5C). We also found that enzalutamide treatment decreases CRAT staining by IHC (Fig. 5D). Although CRAT was not decreased by 3 days of enzalutamide treatment of T47D cells (Supplementary Fig. S4A, (43)), it was decreased at 7 days of treatment (Fig. 5E). This implies that CRAT may be indirectly affected by AR. These data also interestingly revealed that the dominant isoform of CRAT is different between MCF7 and T47D cells. MCF7 predominantly express the smaller isoform whereas T47D predominantly express the larger isoform. Regardless of which CRAT isoform was predominantly expressed, enzalutamide consistently reduced the larger isoform. We also found that CRAT was upregulated coincident with AR under anchorage-independent conditions and that AR knockdown inhibited CRAT protein expression in adherent and anchorage-independent culture (Fig. 5F). Since the effects of CRAT depletion on OXPHOS capacity in other systems have been mixed and because AR inhibition may affect OXPHOS by separate mechanisms, we asked whether CRAT knockdown alone affects ER-mutant BC OXPHOS. CRAT knockdown in MCF7 D538G cells affected primarily the larger CRAT isoform with minimal effect on the smaller, dominant isoform (Fig. 5G). However, CRAT knockdown significantly decreased OXPHOS (Fig. 5H and Supplementary Fig. S4B (43 )) indicating that depletion of the larger isoform is sufficient to induce OXPHOS dysfunction. Interestingly, although both shCRAT constructs demonstrated impressive FAO inhibition, only shCRAT-95 demonstrated substantial inhibition of non-FA substrate oxidation, whereas shCRAT-00 retained most of its non-FA oxidation capacity (Fig. 5I). Together, these data indicate that AR supports CRAT expression and that CRAT promotes OXPHOS in ER-mutant BC.

### CRAT Expression Correlates With AR in ER+ BC Patient Samples

**Figure 6.**
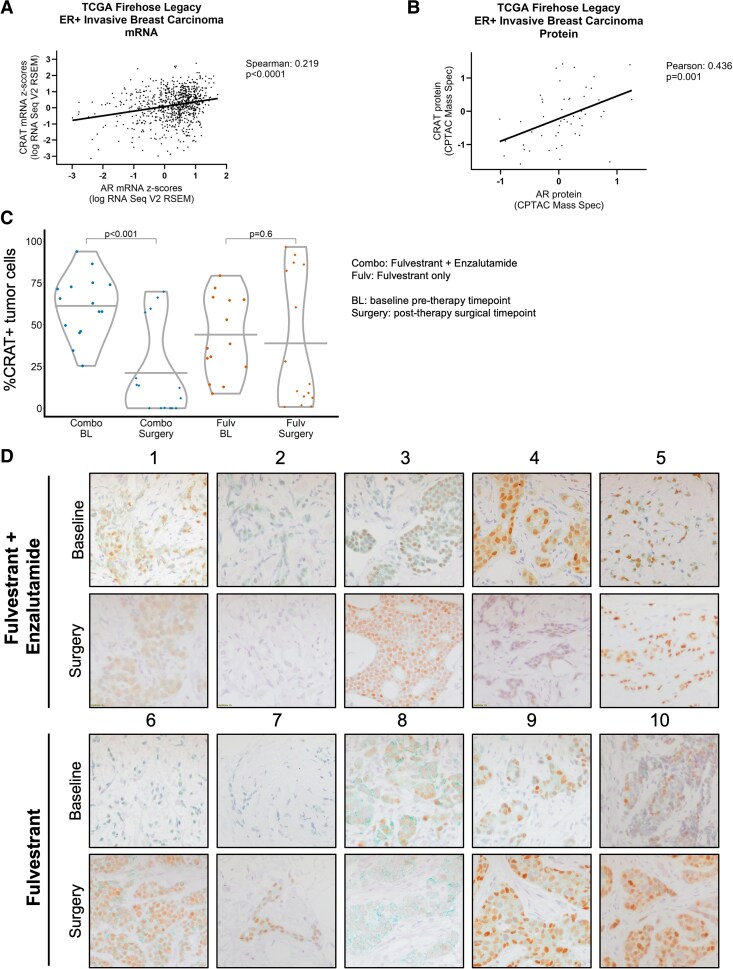
CRAT and AR expression are positively correlated in ER+ BC patient samples. A-B, Correlation analysis of CRAT and AR mRNA (A) or protein (B) expression from TCGA Firehose Legacy ER+ invasive breast carcinoma dataset. A two-tailed Spearman (A) or Pearson (B) correlation analysis was performed. The line through the data points is derived from a simple linear regression analysis. C-D, CRAT is reduced in primary ER+ breast cancer at baseline compared to time of surgery (4 months later) in patients receiving enzalutamide plus fulvestrant, but not fulvestrant only. Patients with ER+ breast cancer enrolled in NCT02955394, a neoadjuvant clinical trial, received either fulvestrant plus enzalutamide or fulvestrant only for 4 months prior to surgery. Tumor biopsies from 5 patients per treatment group at baseline (core needle) and surgery (excisional) were stained for androgen receptor (DAB) followed by CRAT (Vina Green). Percent tumor cell CRAT expression positivity in 3 fields per patient for each timepoint was quantitated by linear mixed-effects model with mean represented by the horizontal line (C). Representative pre-treatment (baseline) and matched post-treatment (surgery) sections from 5 patients per treatment group are demonstrated in (D). Magnification = 400×.

To examine whether *CRAT* and *AR* expression are correlated in larger breast cancer datasets, we queried CRAT and AR mRNA and protein expression in the TCGA Firehose Legacy ER+ invasive breast carcinoma dataset. We found that AR and CRAT expressions are positively correlated on both the protein and mRNA levels (Fig. 6A and 6B). We also asked whether enzalutamide affects CRAT protein expression in primary ER+ BC samples from a neoadjuvant clinical trial our group conducted examining efficacy of the ER degrader fulvestrant alone vs in combination with enzalutamide (52). We found that samples from patients treated for 4 months with fulvestrant and enzalutamide demonstrated decreased CRAT IHC staining compared to baseline, but that samples from patients treated with fulvestrant only demonstrated no change (Fig. 6C and 6D). These data suggest that AR positively regulates CRAT expression even in the broader context of primary ER+ BC, not solely that of ER+ BC that has progressed on AI therapy.

## Discussion

Anti-estrogen therapy, including aromatase inhibition, is an effective standard of care for postmenopausal patients with ER+ BC, but AI-resistant ER-mutant metastatic disease often develops within a decade of treatment. Resistance to AI therapy therefore remains a major barrier to BC treatment and highlights the need for further understanding of tumor biology in this context (E2-deprivation) to devise new therapies. Although the role of AR in ER+ BC is context-dependent, our results demonstrate that AR promotes tumor cell survival in the context of AI-resistant ER-mutant BC. This work also identified that AR promotes OXPHOS and FAO, which are facets of tumor metabolism recently demonstrated to be critical to anti-estrogen/AI-resistant BC. We therefore propose that AR may be clinically useful as a target to achieve tumor-specific OXPHOS inhibition and FDA-approved anti-AR drugs including enzalutamide and the older generation bicalutamide have been shown to be tolerated in women (53, 54). Additionally, our recent clinical results show efficacy of enzalutamide plus fulvestrant therapy compared to fulvestrant alone in the neoadjuvant setting for large primary ER+ BC (52) and for some patients with heavily pretreated ER+ BC (55). These findings emphasize that although we find that AR inhibition strongly inhibited ER+ BC OXPHOS in vitro, the effect in vivo remains to be evaluated particularly with long-term follow-up and may be restricted to a subpopulation identified through further investigation. Furthermore, it would be interesting to determine whether a ketogenic diet increases response to anti-androgen therapy in ESR1 mutant models that metastasize in mice not supplemented with estrogen.

This work explored how AR inhibition affects ER+ BC cell survival in physiologically relevant contexts. We find that AR inhibition promotes palmitate lipotoxicity via apoptosis, which is relevant to at least 2 dominant ER-mutant BC metastatic niches, liver and lung, that have high palmitate abundance (32). Our observation that CD36 and CPT1A are unaffected by enzalutamide suggests that AR antagonism may not suppress cellular uptake or transport of FA, but will inhibit FAO, which should maximize lipotoxicity. We also find that the tumor suppressive effects of AR inhibition are maximized under conditions that require OXPHOS for cellular energetics, including when cells are forced to utilize mitochondrial metabolism of ketones for ATP synthesis. Since we show that AR inhibition does not affect ER-mutant BC glycolysis, but significantly inhibits OXPHOS, we predict that diets like the ketogenic diet that restrict carbohydrates and promote ketone secretion would improve efficacy of AR-targeted therapy.

Prior work in BC preclinical models indicated that metastatic tumor cells are reliant on OXPHOS (29, 56) and that AR is elevated in anchorage-independent BC cells (20). The most direct studies of AR regulation of OXPHOS were in prostate cancer, but the effects of AR on prostate cancer OXPHOS are controversial, as some studies found AR promotes OXPHOS (57, 58), whereas others found AR inhibits OXPHOS (59). Our work finds that AR promotes ER-mutant BC OXPHOS regardless of substrate. RNA sequencing identified a potential mechanism by which AR inhibition disrupts OXPHOS by suppressing CRAT, which is alone sufficient to induce OXPHOS dysfunction. Although we examined the effects of AR inhibition on the transcriptome of ER-mutant BC, it is possible that AR inhibition also perturbs OXPHOS through additional non-genomic functions of AR which might not be captured by RNA sequencing and would require proteomic or phosphoproteomic analysis.

Our study finds that AR promotes CRAT expression. The only previous report linking CRAT to AR demonstrated that CRAT expression and activity are higher in AR-positive PC3 and LNCaP prostate cancer cells compared to AR-negative PNT2 cells (56) but did not perturb AR to demonstrate a causal relationship between AR and CRAT expression. As far as we are aware, ours is the first report using AR perturbation that demonstrates regulation of CRAT by AR. Since our RNA sequencing data demonstrate that enzalutamide inhibits *CRAT* mRNA abundance, it is likely that AR exerts direct or indirect transcriptional regulation of CRAT. We also demonstrate that this regulation likely occurs in all ER+ BC, not only AI-treated samples, and that we can detect a reduction in CRAT expression in patient tumor samples after treatment with clinically tolerated enzalutamide regimens.

We demonstrate that CRAT exists in at least 2 protein isoforms in ER-mutant MCF7 and T47D cells, but that the dominant isoform is different between the 2 cell backgrounds. It is possible that the different isoforms are derived from post-translational processing or alternative reading frames and that these mechanisms differ between MCF7 and T47D cells. Interestingly, acute AR antagonism and CRAT knockdown primarily affect the larger isoform, with minimal effect on the smaller. This could reflect that turnover is greater for the larger isoform than the smaller isoform or that the smaller isoform is somehow protected from genetic suppression of CRAT expression. The isolated effect of acute CRAT knockdown on the larger isoform was informative in that it reveals that suppression of that isoform alone is sufficient to exert significant OXPHOS inhibition. CRAT demonstrates multiple banding patterns in human liver mitochondria and peroxisomes (60), but the significance of these isoforms in BC remains unclear. More work is needed to identify where these isoforms localize and whether they affect OXPHOS differently.

The effects of modulating CRAT expression in other studies are mixed, with some demonstrating CRAT overexpression inhibits ovarian cancer growth and OXPHOS (49), whereas others found that CRAT depletion inhibits OXPHOS (48) and melanoma metastasis (61). However, compound heterozygous inactivating CRAT missense variants were recently reported in a patient with the severe mitochondriopathy Leigh syndrome (62), indicating that total CRAT inhibition by enzymatic inactivation is associated with severe mitochondrial dysfunction in multiple tissue types in vivo. Our work also identifies that AR antagonist- or knockdown-mediated OXPHOS inhibition is coincident with a slight upregulation of mtDNA-encoded mRNA expression, as has been previously reported by CRAT knockdown alone (61). This mtRNA upregulation is not associated with an increase in overall mitochondrial content per cell but may reflect a feedback loop that senses reduced OXPHOS capacity and attempts to upregulate mtDNA gene expression to compensate.

We find that enzalutamide treatment induces overabundance of most even-chain acylcarnitine species, consistent with defective FAO (46), and an endoplasmic reticulum stress transcriptional signature. Recent work by others demonstrated that acylcarnitine overabundance inhibits OXPHOS (63) and long-chain acylcarnitines induce cell death by activating JNK and ERK and inducing endoplasmic reticulum stress (64). These findings suggest that there may be a loop in which AR inhibition induces OXPHOS dysfunction that causes acylcarnitine accumulation that feeds forward to further inhibit OXPHOS and causes endoplasmic reticulum stress-associated cell death.

Our RNA sequencing data revealed a very strong decrease in cell cycle pathway activation, and metabolomics revealed a signature strongly associated with FAO and OXPHOS inhibition. Three of the metabolites that demonstrated the greatest decrease in abundance by enzalutamide treatment were L-aspartate, argininosuccinate, and UDP N-acetylglucosamine. L-aspartate is critical to nucleotide synthesis and is synthesized from TCA-derived oxaloacetate and thus its production is decreased under OXPHOS inhibition. Argininosuccinate and UDP N-acetylglucosamine require aspartate for their synthesis and OXPHOS promotes UDP N-acetylglucosamine synthesis (65). UDP N-acetylglucosamine promotes OXPHOS by modifying mitochondrial proteins through N-acetylglucosamination (66). Thus, the metabolic effects of AR inhibition strongly indicate, and may potentiate, inhibition of OXPHOS that normally promotes nucleotide synthesis important for cell cycle progression. These metabolic changes also coincide with CRAT downregulation, which has been demonstrated to inhibit cell cycle progression (67). These findings may in part explain why cell cycle pathways were the dominant negative signature under enzalutamide treatment.

**Figure 7.**
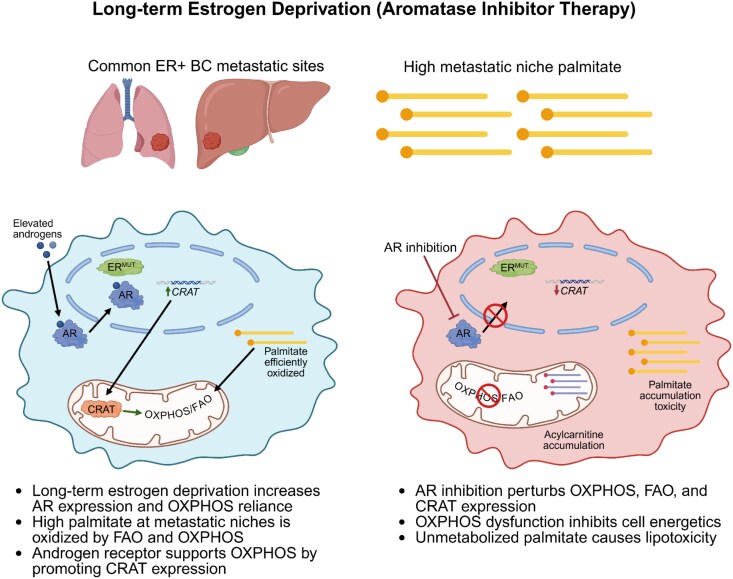
Model of AR promotion of ER-mutant BC growth and OXPHOS by mechanisms including promotion of CRAT expression under LTED. Long-term estrogen deprivation by AI therapy for ER+ BC results in development of AI-resistant ER-mutant BC metastases in the liver and lung, which have high abundance of palmitate. LTED causes upregulation of, and reliance on, OXPHOS and FAO. Elevated androgens from AI therapy activate AR which promotes OXPHOS by mechanisms including upregulation of CRAT expression. Anti-androgen therapy inhibits OXPHOS, FAO, and CRAT expression causing decreased cell growth and cell death from palmitate that is inefficiently oxidized by FAO. Created in BioRender. Sessions, D. (2025)  https://BioRender.com/4dnpsly.

In all, we demonstrate that AR promotes ER-mutant BC growth, OXPHOS, and resistance to palmitate lipotoxicity under conditions mimicking standard-of-care AI therapy under which ER-mutant metastatic breast cancer develops (Fig. 7). One mechanism by which AR promotes OXPHOS is by promoting expression of CRAT. This study adds to a body of literature examining the contexts in which AR supports ER+ BC progression and provides rationale for using anti-androgen therapy in the context of ER-mutant BC, particularly under the context of AI resistance.

## Data Availability

Some or all datasets generated during and/or analyzed during the current study are not publicly available but are available from the corresponding author on reasonable request.

## Abbreviations

AI: aromatase inhibitor
AR: androgen receptors
BC: breast cancer
CRAT: carnitine O-acetyltransferase
DHT: dihydrotestosterone
DMSO: dimethyl sulfoxide
ER: estrogen receptor
ETC: electron transport chain
FA: fatty acid
FAO: fatty acid oxidation
FBS: fetal bovine serum
GSEA: gene set enrichment analysis
LTED: long-term estrogen deprivation
MS: mass spectrometry
mtDNA: mitochondrial DNA
OCR: oxygen consumption capacity
OXPHOS: oxidative phosphorylation
PBS: phosphate-buffered saline
qPCR: quantitative polymerase chain reaction
TBST: Tris-buffered saline with Tween-20

## References

[1] Early Breast Cancer Trialists’ Collaborative Group (EBCTCG) . Aromatase inhibitors versus tamoxifen in early breast cancer: patient-level meta-analysis of the randomised trials. Lancet. 2015;386(10001):1341‐1352. 26211827 10.1016/S0140-6736(15)61074-1

[2] Early Breast Cancer Trialists’ Collaborative Group (EBCTCG) . Aromatase inhibitors versus tamoxifen in premenopausal women with oestrogen receptor-positive early-stage breast cancer treated with ovarian suppression: a patient-level meta-analysis of 7030 women from four randomised trials. Lancet Oncol. 2022;23(3):382‐392. 35123662 10.1016/S1470-2045(21)00758-0 PMC8885431

[3] Takagi K, Miki Y, Nagasaki S, et al Increased intratumoral androgens in human breast carcinoma following aromatase inhibitor exemestane treatment. Endocr Relat Cancer. 2010;17(2):415‐430. 20228125 10.1677/ERC-09-0257

[4] Bleach R, Madden SF, Hawley J, et al Steroid ligands, the forgotten triggers of nuclear receptor action; implications for acquired resistance to endocrine therapy. Clin Cancer Res. 2021;27(14):3980‐3989. 34016642 10.1158/1078-0432.CCR-20-4135 PMC9401529

[5] Gallicchio L, Macdonald R, Wood B, Rushovich E, Helzlsouer KJ. Androgens and musculoskeletal symptoms among breast cancer patients on aromatase inhibitor therapy. Breast Cancer Res Treat. 2011;130(2):569‐577. 21647676 10.1007/s10549-011-1611-2

[6] Caswell-Jin JL, Sun LP, Munoz D, et al Analysis of breast cancer mortality in the US-1975 to 2019. JAMA. 2024;331(3):233‐241. 38227031 10.1001/jama.2023.25881 PMC10792466

[7] Jeselsohn R, Yelensky R, Buchwalter G, et al Emergence of constitutively active estrogen receptor-alpha mutations in pretreated advanced estrogen receptor-positive breast cancer. Clin Cancer Res. 2014;20(7):1757‐1767. 24398047 10.1158/1078-0432.CCR-13-2332 PMC3998833

[8] Toy W, Shen Y, Won H, et al ESR1 ligand-binding domain mutations in hormone-resistant breast cancer. Nat Genet. 2013;45(12):1439‐1445. 24185512 10.1038/ng.2822 PMC3903423

[9] Li S, Shen D, Shao J, et al Endocrine-therapy-resistant ESR1 variants revealed by genomic characterization of breast-cancer-derived xenografts. Cell Rep. 2013;4(6):1116‐1130. 24055055 10.1016/j.celrep.2013.08.022 PMC3881975

[10] Merenbakh-Lamin K, Ben-Baruch N, Yeheskel A, et al D538g mutation in estrogen receptor-alpha: a novel mechanism for acquired endocrine resistance in breast cancer. Cancer Res. 2013;73(23):6856‐6864. 24217577 10.1158/0008-5472.CAN-13-1197

[11] Robinson DR, Wu YM, Vats P, et al Activating ESR1 mutations in hormone-resistant metastatic breast cancer. Nat Genet. 2013;45(12):1446‐1451. 24185510 10.1038/ng.2823 PMC4009946

[12] Martin LA, Ribas R, Simigdala N, et al Discovery of naturally occurring ESR1 mutations in breast cancer cell lines modelling endocrine resistance. Nat Commun. 2017;8(1):1865. 29192207 10.1038/s41467-017-01864-y PMC5709387

[13] Arnesen S, Blanchard Z, Williams MM, et al Estrogen receptor alpha mutations in breast cancer cells cause gene expression changes through constant activity and secondary effects. Cancer Res. 2021;81(3):539‐551. 33184109 10.1158/0008-5472.CAN-20-1171 PMC7854489

[14] Collins LC, Cole KS, Marotti JD, Hu R, Schnitt SJ, Tamimi RM. Androgen receptor expression in breast cancer in relation to molecular phenotype: results from the nurses’ health study. Mod Pathol. 2011;24(7):924‐931. 21552212 10.1038/modpathol.2011.54 PMC3128675

[15] Vera-Badillo FE, Templeton AJ, de Gouveia P, et al Androgen receptor expression and outcomes in early breast cancer: a systematic review and meta-analysis. J Natl Cancer Inst. 2014;106(1):djt319. 24273215 10.1093/jnci/djt319

[16] D'Amato NC, Gordon MA, Babbs B, et al Cooperative dynamics of AR and ER activity in breast cancer. Mol Cancer Res. 2016;14(11):1054‐1067. 27565181 10.1158/1541-7786.MCR-16-0167 PMC5107172

[17] Aceto N, Bardia A, Wittner BS, et al AR expression in breast cancer CTCs associates with bone metastases. Mol Cancer Res. 2018;16(4):720‐727. 29453314 10.1158/1541-7786.MCR-17-0480 PMC5882540

[18] Hickey TE, Selth LA, Chia KM, et al The androgen receptor is a tumor suppressor in estrogen receptor-positive breast cancer. Nat Med. 2021;27(2):310‐320. 33462444 10.1038/s41591-020-01168-7

[19] Garay JP, Park BH. Androgen receptor as a targeted therapy for breast cancer. Am J Cancer Res. 2012;2(4):434‐445. 22860233 PMC3410582

[20] Williams MM, Spoelstra NS, Arnesen S, et al Steroid hormone receptor and infiltrating immune cell Status reveals therapeutic vulnerabilities of ESR1-mutant breast cancer. Cancer Res. 2021;81(3):732‐746. 33184106 10.1158/0008-5472.CAN-20-1200 PMC7854521

[21] Birrell SN, Bentel JM, Hickey TE, et al Androgens induce divergent proliferative responses in human breast cancer cell lines. J Steroid Biochem Mol Biol. 1995;52(5):459‐467. 7748811 10.1016/0960-0760(95)00005-k

[22] Lin HY, Sun M, Lin C, et al Androgen-induced human breast cancer cell proliferation is mediated by discrete mechanisms in estrogen receptor-alpha-positive and -negative breast cancer cells. J Steroid Biochem Mol Biol. 2009;113(3-5):182‐188. 19159686 10.1016/j.jsbmb.2008.12.010

[23] Cochrane DR, Bernales S, Jacobsen BM, et al Role of the androgen receptor in breast cancer and preclinical analysis of enzalutamide. Breast Cancer Res. 2014;16(1):R7. 24451109 10.1186/bcr3599 PMC3978822

[24] De Amicis F, Thirugnansampanthan J, Cui Y, et al Androgen receptor overexpression induces tamoxifen resistance in human breast cancer cells. Breast Cancer Res Treat. 2010;121(1):1‐11. 19533338 10.1007/s10549-009-0436-8 PMC2995248

[25] Hampsch RA, Wells JD, Traphagen NA, et al AMPK activation by metformin promotes survival of dormant ER(+) breast cancer cells. Clin Cancer Res. 2020;26(14):3707‐3719. 32321715 10.1158/1078-0432.CCR-20-0269 PMC7367755

[26] Fiorillo M, Sanchez-Alvarez R, Sotgia F, Lisanti MP. The ER-alpha mutation Y537S confers tamoxifen-resistance via enhanced mitochondrial metabolism, glycolysis and rho-GDI/PTEN signaling: implicating TIGAR in somatic resistance to endocrine therapy. Aging (Albany NY). 2018;10(12):4000‐4023. 30573703 10.18632/aging.101690 PMC6326696

[27] Zinger L, Merenbakh-Lamin K, Klein A, et al Ligand-binding domain-activating mutations of ESR1 rewire cellular metabolism of breast cancer cells. Clin Cancer Res. 2019;25(9):2900‐2914. 30733228 10.1158/1078-0432.CCR-18-1505

[28] El-Botty R, Morriset L, Montaudon E, et al Oxidative phosphorylation is a metabolic vulnerability of endocrine therapy and palbociclib resistant metastatic breast cancers. Nat Commun. 2023;14(1):4221. 37452026 10.1038/s41467-023-40022-5 PMC10349040

[29] Tau S, Chamberlin MD, Yang H, et al Oxidative phosphorylation is a metabolic vulnerability of endocrine therapy-tolerant persister cells in ER+ breast cancer. Cancer Res. 2025;85(6):1145‐1161. 39777474 10.1158/0008-5472.CAN-24-1204 PMC11908958

[30] Zuo Q, Yoo JY, Nelson ER, Sikora MJ, Riggins RB, Madak-Erdogan Z. Co-targeting of metabolism using dietary and pharmacologic approaches reduces breast cancer metastatic burden. NPJ Breast Cancer. 2025;11(1):3. 39809806 10.1038/s41523-024-00715-6 PMC11733225

[31] Ahn S, Park JH, Grimm SL, et al Metabolomic rewiring promotes endocrine therapy resistance in breast cancer. Cancer Res. 2024;84(2):291‐304. 37906431 10.1158/0008-5472.CAN-23-0184 PMC10842725

[32] Altea-Manzano P, Doglioni G, Liu Y, et al A palmitate-rich metastatic niche enables metastasis growth via p65 acetylation resulting in pro-metastatic NF-kappaB signaling. Nat Cancer. 2023;4(3):344‐364. 36732635 10.1038/s43018-023-00513-2 PMC7615234

[33] Wieder N, Fried JC, Kim C, et al FALCON systematically interrogates free fatty acid biology and identifies a novel mediator of lipotoxicity. Cell Metab. 2023;35(5):887‐905.e11. 37075753 10.1016/j.cmet.2023.03.018 PMC10257950

[34] Piccolis M, Bond LM, Kampmann M, et al Probing the global cellular responses to lipotoxicity caused by saturated fatty acids. Mol Cell. 2019;74(1):32‐44.e8. 30846318 10.1016/j.molcel.2019.01.036 PMC7696670

[35] Zhang X, Dang CV. Time to hit pause on mitochondria-targeting cancer therapies. Nat Med. 2023;29(1):29‐30. 36658424 10.1038/s41591-022-02129-y PMC11892798

[36] Robinson MD, McCarthy DJ, Smyth GK. Edger: a bioconductor package for differential expression analysis of digital gene expression data. Bioinformatics. 2010;26(1):139‐140. 19910308 10.1093/bioinformatics/btp616 PMC2796818

[37] Ritchie ME, Phipson B, Wu D, et al Limma powers differential expression analyses for RNA-Sequencing and microarray studies. Nucleic Acids Res. 2015;43(7):e47. 25605792 10.1093/nar/gkv007 PMC4402510

[38] Liberzon A, Subramanian A, Pinchback R, Thorvaldsdottir H, Tamayo P, Mesirov JP. Molecular signatures database (MSigDB) 3.0. Bioinformatics. 2011;27(12):1739‐1740. 21546393 10.1093/bioinformatics/btr260 PMC3106198

[39] Nemkov T, Reisz JA, Gehrke S, Hansen KC, D'Alessandro A. High-throughput metabolomics: isocratic and gradient mass spectrometry-based methods. Methods Mol Biol. 2019;1978:13‐26. 31119654 10.1007/978-1-4939-9236-2_2

[40] Nemkov T, Hansen KC, D'Alessandro A. A three-minute method for high-throughput quantitative metabolomics and quantitative tracing experiments of central carbon and nitrogen pathways. Rapid Commun Mass Spectrom. 2017;31(8):663‐673. 28195377 10.1002/rcm.7834 PMC5364945

[41] Rohani A, Kashatus JA, Sessions DT, Sharmin S, Kashatus DF. Mito hacker: a set of tools to enable high-throughput analysis of mitochondrial network morphology. Sci Rep. 2020;10(1):18941. 33144635 10.1038/s41598-020-75899-5 PMC7642274

[42] Wei L, Gao H, Yu J, et al Pharmacological targeting of androgen receptor elicits context-specific effects in estrogen receptor-positive breast cancer. Cancer Res. 2023;83(3):456‐470. 36469363 10.1158/0008-5472.CAN-22-1016 PMC9896025

[43] Sessions DT, Spoelstra NS, Caino MC, Yu M, Goodspeed A, Richer JK. 2025. Supplemental Figures to “Androgen receptors promote oxidative phosphorylation and resistance to palmitate lipotoxicity in ER-mutant breast cancer”. Figshare. Doi:10.6084/m9.figshare.29247506.v1 PMC12679916 41216931

[44] Liang Y, Han H, Liu L, et al CD36 plays a critical role in proliferation, migration and tamoxifen-inhibited growth of ER-positive breast cancer cells. Oncogenesis. 2018;7(12):98. 30573731 10.1038/s41389-018-0107-x PMC6302092

[45] Listenberger LL, Han X, Lewis SE, et al Triglyceride accumulation protects against fatty acid-induced lipotoxicity. Proc Natl Acad Sci U S A. 2003;100(6):3077‐3082. 12629214 10.1073/pnas.0630588100 PMC152249

[46] El-Gharbawy A, Vockley J. Inborn errors of metabolism with myopathy: defects of fatty acid oxidation and the carnitine shuttle system. Pediatr Clin North Am. 2018;65(2):317‐335. 29502916 10.1016/j.pcl.2017.11.006 PMC6566095

[47] Violante S, Ijlst L, Ruiter J, et al Substrate specificity of human carnitine acetyltransferase: implications for fatty acid and branched-chain amino acid metabolism. Biochim Biophys Acta. 2013;1832(6):773‐779. 23485643 10.1016/j.bbadis.2013.02.012

[48] Song MJ, Park CH, Kim H, et al Carnitine acetyltransferase deficiency mediates mitochondrial dysfunction-induced cellular senescence in dermal fibroblasts. Aging Cell. 2023;22(11):e14000. 37828898 10.1111/acel.14000 PMC10652321

[49] Zhang Z, Zhao S, Lv X, et al CRAT downregulation promotes ovarian cancer progression by facilitating mitochondrial metabolism through decreasing the acetylation of PGC-1alpha. Cell Death Discov. 2025;11(1):15. 39828731 10.1038/s41420-025-02294-2 PMC11743791

[50] Yu G, Cheng CJ, Lin SC, et al Organelle-derived acetyl-CoA promotes prostate cancer cell survival, migration, and metastasis via activation of calmodulin kinase II. Cancer Res. 2018;78(10):2490‐2502. 29535221 10.1158/0008-5472.CAN-17-2392 PMC6556391

[51] Sun X, Lu Q, Yegambaram M, et al TGF-beta1 attenuates mitochondrial bioenergetics in pulmonary arterial endothelial cells via the disruption of carnitine homeostasis. Redox Biol. 2020;36:101593. 32554303 10.1016/j.redox.2020.101593 PMC7303661

[52] Elias AD, Staley AW, Fornier M, et al Clinical and immune responses to neoadjuvant fulvestrant with or without enzalutamide in ER+/Her2- breast cancer. NPJ Breast Cancer. 2024;10(1):88. 39368973 10.1038/s41523-024-00697-5 PMC11455938

[53] Schwartzberg LS, Yardley DA, Elias AD, et al A phase I/Ib study of enzalutamide alone and in combination with endocrine therapies in women with advanced breast cancer. Clin Cancer Res. 2017;23(15):4046‐4054. 28280092 10.1158/1078-0432.CCR-16-2339

[54] Krop I, Abramson V, Colleoni M, et al A randomized placebo controlled phase II trial evaluating exemestane with or without enzalutamide in patients with hormone receptor-positive breast cancer. Clin Cancer Res. 2020;26(23):6149‐6157. 32988969 10.1158/1078-0432.CCR-20-1693

[55] Elias AD, Spoelstra NS, Staley AW, et al Phase II trial of fulvestrant plus enzalutamide in ER+/HER2- advanced breast cancer. NPJ Breast Cancer. 2023;9(1):41. 37210417 10.1038/s41523-023-00544-z PMC10199936

[56] Davis RT, Blake K, Ma D, et al Transcriptional diversity and bioenergetic shift in human breast cancer metastasis revealed by single-cell RNA sequencing. Nat Cell Biol. 2020;22(3):310‐320. 32144411 10.1038/s41556-020-0477-0

[57] Bader DA, Hartig SM, Putluri V, et al Mitochondrial pyruvate import is a metabolic vulnerability in androgen receptor-driven prostate cancer. Nat Metab. 2019;1(1):70‐85. 31198906 10.1038/s42255-018-0002-y PMC6563330

[58] Lee YG, Nam Y, Shin KJ, et al Androgen-induced expression of DRP1 regulates mitochondrial metabolic reprogramming in prostate cancer. Cancer Lett. 2020;471:72‐87. 31838085 10.1016/j.canlet.2019.12.017

[59] Bajpai P, Koc E, Sonpavde G, Singh R, Singh KK. Mitochondrial localization, import, and mitochondrial function of the androgen receptor. J Biol Chem. 2019;294(16):6621‐6634. 30792308 10.1074/jbc.RA118.006727 PMC6484137

[60] Bloisi W, Colombo I, Garavaglia B, Giardini R, Finocchiaro G, Didonato S. Purification and properties of carnitine acetyltransferase from human liver. Eur J Biochem. 1990;189(3):539‐546. 2351134 10.1111/j.1432-1033.1990.tb15520.x

[61] Lasheras-Otero I, Feliu I, Maillo A, et al The regulators of peroxisomal acyl-carnitine shuttle CROT and CRAT promote metastasis in melanoma. J Invest Dermatol. 2023;143(2):305‐316.e305. 36058299 10.1016/j.jid.2022.08.038

[62] Laera L, Punzi G, Porcelli V, et al CRAT missense variants cause abnormal carnitine acetyltransferase function in an early-onset case of Leigh syndrome. Hum Mutat. 2020;41(1):110‐114. 31448845 10.1002/humu.23901

[63] Liepinsh E, Makrecka-Kuka M, Volska K, et al Long-chain acylcarnitines determine ischaemia/reperfusion-induced damage in heart mitochondria. Biochem J. 2016;473(9):1191‐1202. 26936967 10.1042/BCJ20160164

[64] McCoin CS, Knotts TA, Ono-Moore KD, Oort PJ, Adams SH. Long-chain acylcarnitines activate cell stress and myokine release in C2C12 myotubes: calcium-dependent and -independent effects. Am J Physiol Endocrinol Metab. 2015;308(11):E990‐E1000. 25852008 10.1152/ajpendo.00602.2014 PMC4451287

[65] Cao J, Li M, Liu K, et al Oxidative phosphorylation safeguards pluripotency via UDP-N-acetylglucosamine. Protein Cell. 2023;14(5):376‐381. 37155316 10.1093/procel/pwac009 PMC10166152

[66] Dontaine J, Bouali A, Daussin F, et al The intra-mitochondrial O-GlcNAcylation system rapidly modulates OXPHOS function and ROS release in the heart. Commun Biol. 2022;5(1):349. 35414690 10.1038/s42003-022-03282-3 PMC9005719

[67] Brunner S, Kramar K, Denhardt DT, Hofbauer R. Cloning and characterization of murine carnitine acetyltransferase: evidence for a requirement during cell cycle progression. Biochem J. 1997;322(Pt 2):403‐410. 9065756 10.1042/bj3220403 PMC1218205

